# CO_2_ Adsorption on PtCu Sub-Nanoclusters Deposited on Pyridinic N-Doped Graphene: A DFT Investigation

**DOI:** 10.3390/ma14247619

**Published:** 2021-12-10

**Authors:** Fernando Montejo-Alvaro, Diego González-Quijano, Jorge A. Valmont-Pineda, Hugo Rojas-Chávez, José M. Juárez-García, Dora I. Medina, Heriberto Cruz-Martínez

**Affiliations:** 1 Tecnológico Nacional de México, Instituto Tecnológico del Valle de Etla, Abasolo S/N, Barrio del Agua Buena, Santiago Suchilquitongo, Oaxaca 68230, Mexico; moaf1217@gmail.com; 2 Centro de Ciencias de la Ingeniería, Universidad Autónoma de Aguascalientes Campus sur, Av. Prolongación Mahatma Ghandi 6601, Colonia el Gigante 20340, Aguascalientes, Mexico; diegoxjgq@gmail.com; 3 Tecnológico Nacional de México, Instituto Tecnológico del Istmo, Panamericana 821, 2da., Juchitán de Zaragoza, Oaxaca 70000, Mexico; jorge.vp@itistmo.tecnm.mx; 4 Tecnológico Nacional de México, Instituto Tecnológico de Tláhuac II, Camino Real 625, Tláhuac, Ciudad de México 13508, Mexico; rojas_hugo@ittlahuac2.edu.mx; 5 Universidad Tecnológica del Estado de Querétaro, Av. Pie de la Cuesta 2501, Nacional, Santiago de Querétaro, Querétaro 76148, Mexico; josman_jg@yahoo.com.mx; 6 Tecnologico de Monterrey, School of Engineering and Sciences, Atizapan de Zaragoza 52926, Estado de Mexico, Mexico

**Keywords:** CO_2_ adsorption, adsorption energy, charge transfer, stability

## Abstract

To reduce the CO_2_ concentration in the atmosphere, its conversion to different value-added chemicals plays a very important role. Nevertheless, the stable nature of this molecule limits its conversion. Therefore, the design of highly efficient and selective catalysts for the conversion of CO_2_ to value-added chemicals is required. Hence, in this work, the CO_2_ adsorption on Pt_4-x_Cu_x_ (x = 0–4) sub-nanoclusters deposited on pyridinic N-doped graphene (PNG) was studied using the density functional theory. First, the stability of Pt_4-x_Cu_x_ (x = 0–4) sub-nanoclusters supported on PNG was analyzed. Subsequently, the CO_2_ adsorption on Pt_4-x_Cu_x_ (x = 0–4) sub-nanoclusters deposited on PNG was computed. According to the binding energies of the Pt_4-x_Cu_x_ (x = 0–4) sub-nanoclusters on PNG, it was observed that PNG is a good material to stabilize the Pt_4-x_Cu_x_ (x = 0–4) sub-nanoclusters. In addition, charge transfer occurred from Pt_4-x_Cu_x_ (x = 0–4) sub-nanoclusters to the PNG. When the CO_2_ molecule was adsorbed on the Pt_4-x_Cu_x_ (x = 0–4) sub-nanoclusters supported on the PNG, the CO_2_ underwent a bond length elongation and variations in what bending angle is concerned. In addition, the charge transfer from Pt_4-x_Cu_x_ (x = 0–4) sub-nanoclusters supported on PNG to the CO_2_ molecule was observed, which suggests the activation of the CO_2_ molecule. These results proved that Pt_4-x_Cu_x_ (x = 0–4) sub-nanoclusters supported on PNG are adequate candidates for CO_2_ adsorption and activation.

## 1. Introduction

Due to human activity associated with the usage of fossil fuels and industrialization, the concentration of CO_2_ in the atmosphere has increased considerably. It is causing environmental problems such as the greenhouse effect, global warming, and climate change among others [1,2,3]. Therefore, in order to reduce the CO_2_ concentration in the atmosphere, various investigations and technologies are being developed such as the CO_2_ sequestration process [4,5], and CO_2_ conversion into different value-added chemicals is another strategy widely used [6,7,8]. Nevertheless, the stable nature of the CO_2_ molecule limits its conversion [9,10]. Therefore, the design of highly efficient and selective catalysts for the conversion of CO_2_ into value-added chemicals is required.

Currently, many catalysts have been designed for CO_2_ conversion into value-added chemical products, among them the transition metal nanoparticles-based catalysts can be highlighted [11,12]. However, more recently, it has been documented that alloy catalysts have more outstanding catalytic activities than monometallic nanoparticles for CO_2_ conversion [13,14]. Among the different bimetallic catalysts studied, PtCu nanoparticles have become very important because they present good catalytic properties for the CO_2_ conversion [15,16,17]. For example, Cu–Pt alloy nanocubes with a relatively broad range of composition ratios were synthesized and evaluated for CO_2_ electroreduction reaction [15]. It was found that the Cu–Pt alloys exhibit compositional-dependent activities towards CO_2_ electroreduction. In another study, Cu–Pt nanocrystals with different amounts of Cu and Pt were prepared and evaluated for CO_2_ electroreduction reaction [16]. Guo and coworkers highlighted the importance of the compositional effect of Cu–Pt nanocrystals on their catalytical activities in what CO_2_ electroreduction is concerned. In addition, it was demonstrated that the Cu–Pt (3:1) nanocrystals exhibited the highest activity and faradaic efficiency in the CO_2_ electroreduction reaction among all the as-prepared Cu–Pt samples. Recently, a density functional theory (DFT) study of CO_2_ adsorption on Cu_4-x_Pt_x_ (x = 0–4) clusters was performed [18]. It was computed that the gas phase linear CO_2_ molecule was deformed upon adsorption, with its bend angle varying from about 134° to 145°, which could favor the CO_2_ dissociation. It can be inferred from these studies that Cu–Pt alloys are good candidates for the conversion of CO_2_ to value-added products.

However, it is well known that metallic nanoparticles tend to agglomerate due to their high surface energies, which in turn involves the coarsening of larger particles from those of smaller size [19,20]. Therefore, to overcome the agglomeration problems, it is necessary to disperse or support these nanoparticles on materials with high surface area. To this end, graphene is considered a good support material due to its high specific surface area, excellent electrical conductivity, and resistance to corrosion [21,22], however, this material has a limited chemical reactivity [23]. Consequently, various approaches have been implemented to improve its activity, e.g., functionalization and doping among others [24,25,26]. Specifically, among the different dopants used to modify graphene reactivity, pyridinic-type N doping can be highlighted because it enhances both the stability and reactivity of metallic nanoparticles [27,28]. Nowadays, there is a sizeable number of theoretical studies that analyze the stability and reactivity of metal nanoparticles supported on pyridinic N-doped graphene (PNG) [29,30,31]. These studies show the potential of PNG to improve not only the stability, but also the reactivity of metal nanoparticles for different applications [29,30,31].

According to the literature, the reactivity and stability of Pt_4-x_Cu_x_ (x = 0–4) clusters supported on PNG substrate was investigated using the DFT [32]. It was demonstrated that Pt–Cu nanoparticles supported on PNG are good candidates to adsorb the glyphosate molecule and PNG stabilized the Pt–Cu nanoparticles as well [32]. However, to the best of our knowledge, there are no theoretical studies on CO_2_ adsorption on Pt–Cu clusters supported on PNG using the DFT calculations. Therefore, in this work, the CO_2_ adsorption on Pt–Cu sub-nanoclusters deposited on PNG was studied using the DFT calculations. In order to achieve this goal, the most stable interaction between the Pt_4-x_Cu_x_ (x = 0–4) sub-nanoclusters and the PNG was investigated. Furthermore, DFT calculations were used to bring light into the CO_2_ adsorption on Pt_4-x_Cu_x_ (x = 0–4) sub-nanoclusters deposited on PNG.

## 2. Computational Methodology

All calculations were carried out within the DFT implemented in the ORCA 5.0.0 package [33]. All the electronic structure calculations were addressed through the revised Perdew–Burke–Ernzerhof exchange correlation functional (revPBE) [34]. For the C, H, N, and O atoms, the Ahlrichs basis sets def2-SVP were used for the calculations and def2-TZVP for the Cu atoms [35], whereas the Pt ones were treated using the basis set LANL2DZ for effective core potentials [36]. The convergence tolerances for geometry optimization were energy change = 5 × 10^−6^ Eh, max. gradient = 3 × 10^−4^ Eh/Bohr, rms gradient = 1 × 10^−4^ Eh/Bohr, max. displacement = 4 × 10^−3^ Bohr, and rms displacement = 2 × 10^−3^ Bohr.

**Figure 1:**
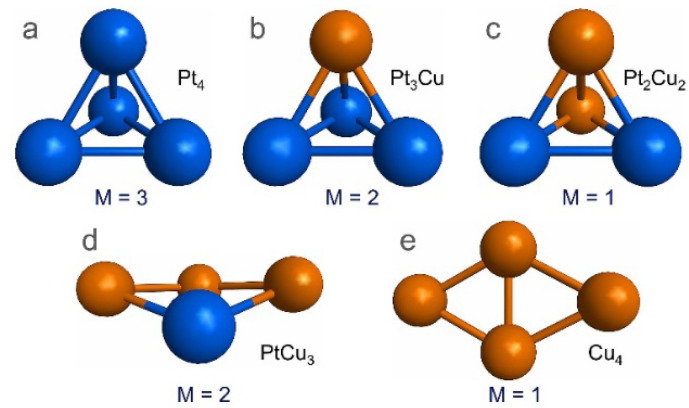
Structures and spin multiplicity (M) of Pt_4-x_Cu_x_ (x = 0–4) sub-nanoclusters. (**a**) Pt_4_, (**b**) Pt_3_Cu, (**c**) Pt_2_Cu_2_, (**d**) PtCu_3_, and (**e**) Cu_4_.

To investigate the stability of Pt_4-x_Cu_x_ (x = 0–4) sub-nanoclusters on PNG, the most stable structures for the Pt_4-x_Cu_x_ (x = 0–4) sub-nanoclusters were obtained from a previous study [18]. However, it is worth highlighting that these structures were reoptimized employing the methodology used in this study, which are depicted in Figure 1.

**Figure 2:**
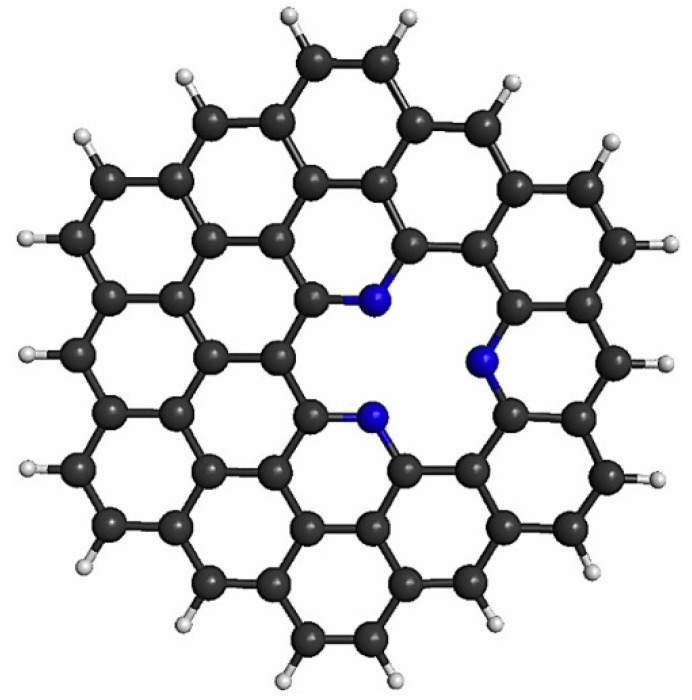
Pyridinic N_3_-doped graphene structure.

The pyridinic-type N doping can be located anywhere on the graphene (e.g., edge or center). Here, we used graphene as the support material, therefore, the doping was localized in the center of the graphene. In this case, different numbers of nitrogen atoms (e.g., 1, 2, or 3) can be used. In this work, we used pyridinic-type doping with three N atoms, as it has been a widely used structure [29,30,31,37]. In this sense, circumcoronene (C_54_H_18_) was used as model of graphene. To obtain the PNG structure, a C atom was removed from the center of the graphene to create a vacancy, then the hanging C atoms were replaced by N ones, as shown in Figure 2. 

The binding energies (E_b_) between the Pt_4-x_Cu_x_ (x = 0–4) sub-nanoclusters and the PNG were calculated as follows:

(1) $$E_{b}=E_{\mathrm{sub}-\mathrm{nanocluster}/\mathrm{PNG}}-\left(E_{\mathrm{sub}-\mathrm{nanocluster}}+E_{\mathrm{PNG}}\right)$$

where $E_{\mathrm{sub}-\mathrm{nanocluster}/\mathrm{PNG}}$, $E_{\mathrm{sub}-\mathrm{nanocluster}}$, and $E_{\mathrm{PNG}}$ are the energies of the Pt_4-x_Cu_x_ (x = 0–4) sub-nanoclusters deposited on PNG, Pt_4-x_Cu_x_ (x = 0–4) sub-nanoclusters, and the PNG structure, respectively.

The adsorption energies (E_ads_) of CO_2_ on Pt_4-x_Cu_x_ (x = 0–4) sub-nanoclusters deposited on PNG were obtained as:

(2) $$E_{\mathrm{ads}}=E_{\mathrm{sub}-\mathrm{nanocluster}/\mathrm{PNG}+{\mathrm{CO}}_{2}}-\left(E_{\mathrm{sub}-\mathrm{nanocluster}/\mathrm{PNG}}+E_{{\mathrm{CO}}_{2}}\right)$$

where $E_{\mathrm{sub}-\mathrm{nanocluster}/\mathrm{PNG}+{\mathrm{CO}}_{2}}$ is the energy of CO_2_ adsorbed on Pt_4-x_Cu_x_ (x = 0–4) sub-nanoclusters deposited on PNG, while $E_{\mathrm{sub}-\mathrm{nanocluster}/\mathrm{PNG}}$ and $E_{{\mathrm{CO}}_{2}}$ are the energies as a single point calculation of the free-standing Pt_4-x_Cu_x_ (x = 0–4) sub-nanoclusters supported on PNG and the CO_2_ molecule from the optimized structure of the Pt_4-x_Cu_x_ (x = 0–4)/PNG+CO_2_ composite, respectively.

To analyze the molecular interactions of the sub-nanoclusters supported on PNG and the CO_2_ adsorption over Pt_4-x_Cu_x_ (x = 0–4) sub-nanoclusters deposited on PNG, the Quantum Theory of Atoms in Molecules (QTAIM) developed by Bader was employed for the charge transfer analyses; to this end, the Multiwfn program was used [38].

## 3. Results

### 3.1. Stability of Pt_4-x_Cu_x_ (x = 0–4) Sub-Nanoclusters on PNG

**Figure 3:**
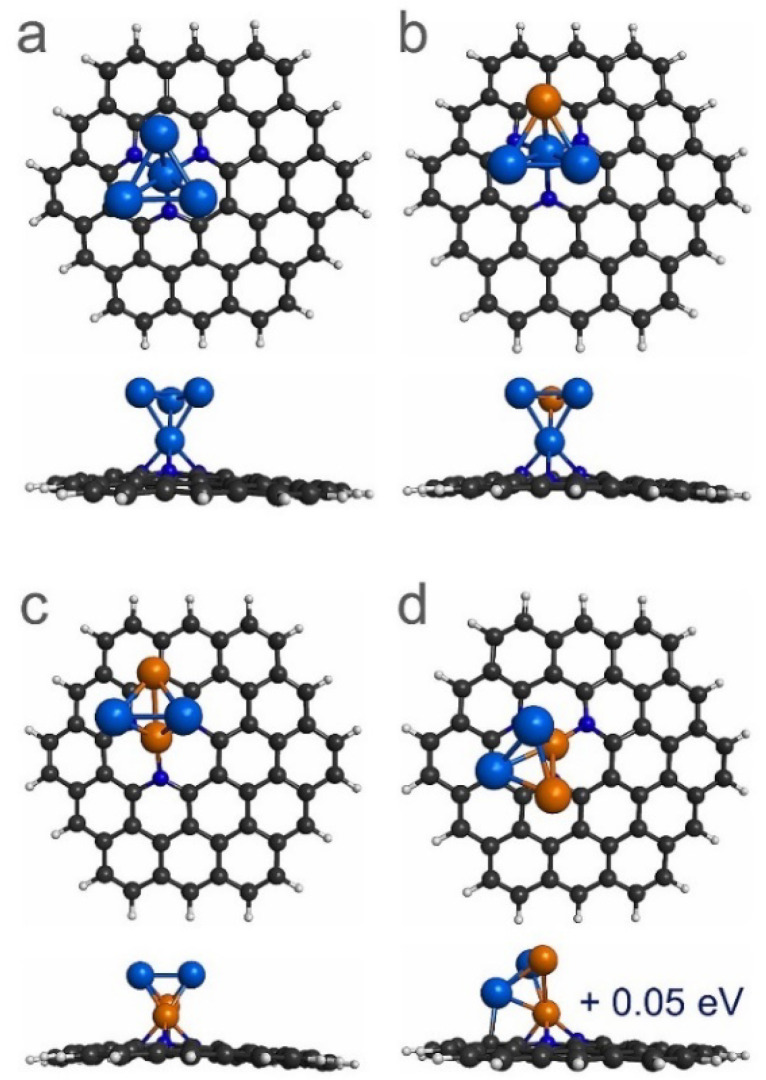
Top and side views of the most stable configurations of the adsorption of Pt_4-x_Cu_x_ (x = 0–2) sub-nanoclusters on PNG. (**a**) Pt_4_/PNG, (**b**) Pt_3_Cu/PNG, and (**c**,**d**) Pt_2_Cu_2_/PNG. Note that (**d**) is a quasi-degenerated state of the Pt_2_Cu_2_/PNG system with a difference in energy of 0.05 eV.

**Figure 4:**
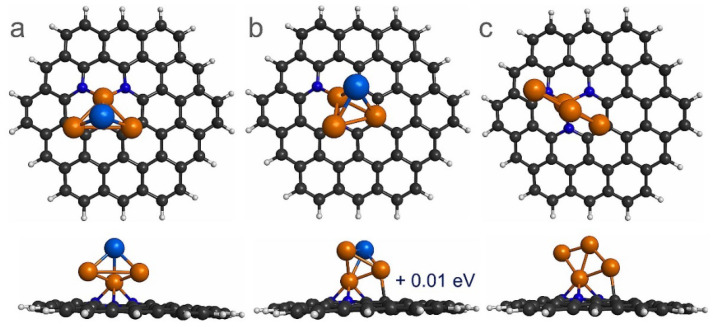
Top and side views of the most stable adsorption sites of Pt_4-x_Cu_x_ (x = 3 and 4) sub-nanoclusters on PNG. (**a**,**b**) PtCu_3_/PNG and (**c**) Cu_4_/PNG. Note that (**b**) is a quasi-degenerated state of the PtCu_3_/PNG system with a difference in energy of 0.01 eV.

The most stable interaction between Pt_4-x_Cu_x_ (n = 0–4) sub-nanoclusters and PNG was determined using several configurations. Figure 3 and Figure 4 illustrate the most stable interactions between the Pt_4-x_Cu_x_ (n = 0–4) sub-clusters and PNG. It was found that the most stable interaction between the Pt_4_ sub-cluster and the PNG was with a Pt atom trapped in the vacancy of the PNG, which is consistent with a previous study reported in literature [39]. It is also investigated that the most stable interaction between the Pt_3_Cu sub-nanocluster and PNG is with a Pt atom trapped in the vacancy of the PNG. For the case of Pt_2_Cu_2_ sub-nanocluster deposited on PNG, two isoenergetic structures were found as the most stable structures, see Figure 3c,d. In the first structure, the interaction occurred with two Cu atoms joined with PNG, where one of the atoms is anchored in the vacancy, while in another structure located at only 0.05 eV above the most stable structure, the interaction is with one atom of Cu and one of Pt, in this case the Cu atom is anchored into the vacancy.

For PtCu_3_ sub-nanoclusters supported on PNG, two isoenergetic configurations were also computed as the most stable structures, see Figure 4a,b. In the case of the most stable interaction, it is observed that the interaction between the PtCu_3_ sub-nanoclusters and PNG occurred with three Cu atoms (Figure 4a), while in another structure, the interaction between the sub-nanocluster and the PNG occurred via two Cu atoms, see Figure 4b. Finally, for Cu_4_ sub-nanocluster deposited on PNG, two Cu atoms interacted with the PNG. In addition, the E_b_ between the Pt_4-x_Cu_x_ (x = 0–4) sub-nanoclusters and the PNG were calculated, see Table 1. It is observed that E_b_ are substantially higher than those reported in previous findings for Pt-based sub-nanoclusters supported on pristine graphene [40,41]. Therefore, it can be inferred that PNG is a good support material for Pt-based nanoclusters. In addition, the calculated E_b_ between the Pt_4_ and the PNG is −3.61 eV, which is similar to that reported in the literature with a value of −4.40 eV [39].

The interaction between the Pt_4-x_Cu_x_ (x = 0–4) sub-nanoclusters and PNG was further investigated by the QTAIM charge transfer, see Table 1. The results suggest that Pt_4-x_Cu_x_ (x = 0–4) sub-nanoclusters transfer charge to the PNG structure since these ended with a total positive charge, which can be attributed to the large electronegativity of the N atoms. Furthermore, it is observed that as the content of Cu in the sub-nanoclusters increases, the charge transfer from sub-nanoclusters to the PNG tends to increase as well, which can be attributed to the low electronegativity of the Cu atoms.

### 3.2. CO_2_ Adsorption on Pt_4-x_Cu_x_ (x = 0–4) Sub-Nanoclusters Deposited on PNG

**Figure 5:**
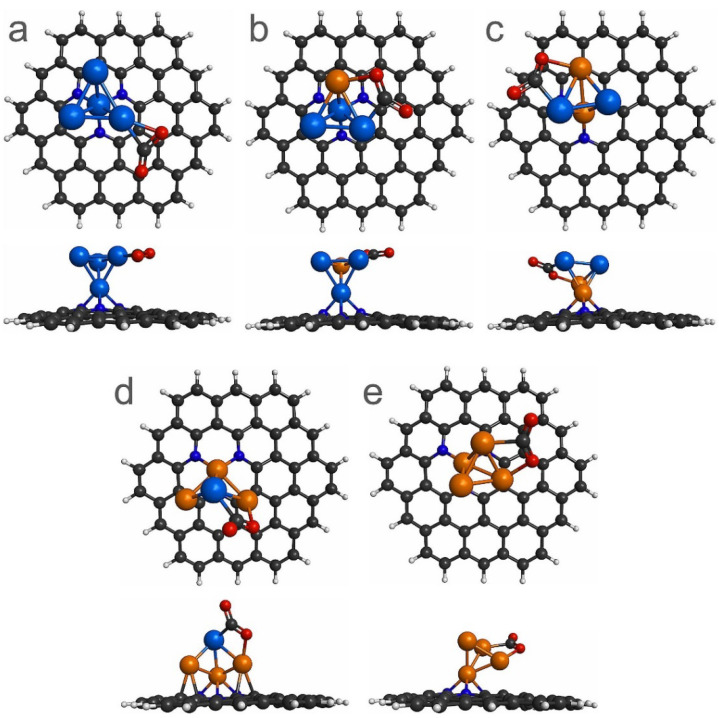
Top and side views of the most stable CO_2_ adsorption sites on the sub-nanoclusters supported on the PNG: (**a**) Pt_4_, (**b**) Pt_3_Cu, (**c**) Pt_2_Cu_2_, (**d**) PtCu_3_, and (**e**) Cu_4_.

To analyze the adsorption and activation of the CO_2_ molecule on the Pt_4-x_Cu_x_ (x = 0–4) sub-nanoclusters deposited on PNG, the CO_2_ adsorption energy, CO_2_ bond elongation, CO_2_ bending angle, and charge transfer from sub-nanoclusters supported PNG to CO_2_ were used as indicators of an effective CO_2_ dissociation process [42,43]. To obtain the most stable interaction between the CO_2_ and sub-nanoclusters supported PNG, several modes (e.g., top, bridge, and hollow) of CO_2_ adsorption on sub-nanoclusters supported on PNG were investigated. In Figure 5, the most stable CO_2_ adsorption on the Pt_4-x_Cu_x_ (x = 0–4) sub-nanoclusters supported on the PNG is reported. The results show that the CO_2_ molecule is deformed when it is adsorbed on the Pt_4-x_Cu_x_ (x = 0–4) sub-nanoclusters supported on PNG (Figure 5), giving way to a bending angle from 135.86° up to 141.25°, see Table 2. Similar results were obtained when the CO_2_ molecule was adsorbed on Cu_4-x_Pt_x_ (x = 0–4) clusters [18]. In addition, it can be observed that the CO_2_ is adsorbed side-on type on Pt_4-x_Cu_x_ (x = 1–4)/PNG composites, whereas for the Pt_4_/PNG composite the CO_2_ molecule is bonded with a Pt atom. The type of CO_2_ adsorption on Pt_4-x_Cu_x_ (x = 1–4)/PNG composites is like those computed on Cu_4-x_Pt_x_ (x = 0–4) clusters [18]. To estimate the E_ads_ between the CO_2_ molecule and the Pt_4-x_Cu_x_ (x = 0–4) sub-nanoclusters deposited on the PNG, the E_ads_ were calculated using Equation (2). It is observed that CO_2_ presented a chemisorption on Pt_4-x_Cu_x_ (x = 0–4) sub-nanoclusters deposited on PNG, since in all cases the E_ads_ were higher than 1 eV. In addition, the CO_2_ molecule is adsorbed stronger on bimetallic Pt_4-x_Cu_x_ (x = 1–4) sub-nanoclusters deposited on PNG than on Pt_4_ sub-nanocluster supported on PNG, which can be attributed to the presence of Cu atoms in bimetallic sub-nanoclusters. Moreover, an elongation of the average C-O bond length is observed when the CO_2_ is adsorbed on Pt_4-x_Cu_x_ (x = 0–4) sub-nanoclusters deposited on PNG (Table 2). It is worth noting that the free CO_2_ presents an average C-O bond length of 1.20 Å. Considering the bond length elongation and the bending angle of the CO_2_ molecule adsorbed on Pt_4-x_Cu_x_ (x = 0–4) sub-nanoclusters deposited on PNG, it is observed that there is an activation of the CO_2_ molecule, which suggests that less energy is required to achieve the dissociation of this molecule. Finally, when CO_2_ is adsorbed on PtCu_3_ and Cu_4_ sub-nanoclusters deposited on PNG, the structures of the PtCu_3_ and Cu_4_ sub-nanoclusters presented a deformation. For instance, the structure of the Cu_4_ sub-nanocluster changes from planar to tetrahedral.

Finally, Table 2 shows the charge transfer between the CO_2_ molecule and the Pt_4-x_Cu_x_ (x = 0–4) sub-nanoclusters supported on the PNG. The total charge of the CO_2_ molecule resulted in negative values for all the systems studied, which indicated that the CO_2_ molecule gained charge after the adsorption. Furthermore, it is observed that as the Cu content in the sub-nanoclusters increases, the charge transfer from the sub-nanoclusters supported on PNG to CO_2_ molecule tends to increase as well, which can be attributed to the low electronegativity of the Cu atoms. Moreover, it is found that the charge transfer plays a significant role in the activation of the CO_2_ molecule [42,43].

## 4. Conclusions

The CO_2_ adsorption on the Pt_4-x_Cu_x_ (x = 0–4) sub-nanoclusters deposited on PNG was studied using the density functional theory. To the best of our knowledge, this is the first study on the CO_2_ adsorption on the Pt_4-x_Cu_x_ (x = 0–4) sub-nanoclusters supported on PNG. First, the stability of the Pt_4-x_Cu_x_ (x = 0–4) sub-nanoclusters supported on PNG was analyzed. The results revealed that PNG enhanced the stability of the Pt_4-x_Cu_x_ (x = 0–4) sub-nanoclusters. After, the CO_2_ adsorption on the Pt_4-x_Cu_x_ (x = 0–4) sub-nanoclusters deposited on PNG was computed. Numerous indicators such as E_ads_, average bond length elongation, angle bending, and charge transfer were used to characterize the CO_2_ interaction on the proposed systems. When the CO_2_ molecule was adsorbed on the Pt_4-x_Cu_x_ (x = 0–4) sub-nanoclusters supported on the PNG, the CO_2_ underwent both bond length elongation and bending angle. In addition, the charge transfer from the Pt_4-x_Cu_x_ (x = 0–4) sub-nanoclusters supported on PNG to the CO_2_ molecule was observed. The results obtained with those indicators suggest that the activation of the CO_2_ molecule took place. Therefore, the Pt_4-x_Cu_x_ (x = 0–4) sub-nanoclusters supported on PNG are suitable candidates for the CO_2_ adsorption and activation.

## Figures and Tables

**Table 1:** Binding energies (E_b_) and charge transfer between the Pt_4-x_Cu_x_ (x = 0–4) sub-nanoclusters and the PNG.

System	E_b_ (eV)	QTAIM Charge (e)
Pt_4_/PNG	−3.61	0.23
Pt_3_Cu/PNG	−3.01	0.26
Pt_2_Cu_2_/PNG	−2.65	0.52
PtCu_3_/PNG	−3.26	0.69
Cu_4_/PNG	−2.44	0.57

**Table 2:** Properties of the CO_2_ adsorption on the Pt_4-x_Cu_x_ (x = 0–4) sub-nanoclusters supported on PNG.

System	E_ads_ (eV)	Charge Transfer Toward CO_2_ (*e*)	Average CO_2_ Bond Length (Å)	Bending Angle of CO_2_ (°)
CO_2_/Pt_4_/PNG	−1.06	−0.37	1.24	141.25
CO_2_/Pt_3_Cu/PNG	−2.21	−0.42	1.24	140.03
CO_2_/Pt_2_Cu_2_/PNG	−2.34	−0.44	1.25	139.32
CO_2_/PtCu_3_/PNG	−2.48	−0.46	1.25	135.86
CO_2_/Cu_4_/PNG	−1.81	−0.58	1.24	138.27

## Data Availability

Not applicable.
